# Violence and Healthcare in Ecuador: Challenges, Responses, and System Resilience

**DOI:** 10.3389/phrs.2025.1607358

**Published:** 2025-02-18

**Authors:** Edy Quizhpe, Karina Pazmiño, Francisco Rodriguez-Lanfranco, Patricia Cueva

**Affiliations:** ^1^ Department Epidemiology and Global Health, Umea University, Umeå, Sweden; ^2^ Comprehensive Geriatric Hospital, Ministry of Health, Quito, Ecuador; ^3^ Medicine Faculty, Universidad San Francisco de Quito, Quito, Ecuador; ^4^ Medicine Faculty, Pontificia Universidad Católica, Quito, Ecuador; ^5^ Medicine Faculty, Universidad de las Américas, Quito, Ecuador

**Keywords:** Ecuador, conflict, health services, crisis, Latin America

In recent years, Ecuador has experienced a notable escalation in violence, presenting a substantial challenge for the public health system. This rise can be attributed to the proliferation of criminal organizations, notably those engaged in drug trafficking, which has resulted in the erosion of state security and regulatory mechanisms, coupled with a surge in governmental corruption [1, 2]. These conditions have further compounded preexisting social dilemmas such as poverty, gender-based violence, and inequality [3].

The healthcare system has not been immune to these effects. Numerous incidents over several years have highlighted deficiencies in the administration of public healthcare services, particularly within the Ministry of Public Health (MoPH) and the National Social Security Institute (Instituto Ecuatoriano de Seguridad Social in Spanish). These incidents often involved issues with the attainment of healthcare services, including overpricing and poor quality of medicines and medical supplies [4]. Furthermore, there has been notable turnover within healthcare management roles at both the national and local levels, frequently influenced by political favoritism in endeavors to improve governance. Additionally, healthcare institutions have encountered challenges stemming from the presence of violent factions, particularly in coastal regions, which have posed risks for both patients and personnel.

In response to these challenges, the Ministry of Health has instituted several measures aimed at safeguarding healthcare professionals, facilities, and users amidst the escalating violence. One notable initiative is the implementation of the Silver Code protocol, introduced in 2022, which delineates inter-institutional procedures designed to ensure the safety of all individuals accessing healthcare institutions, whether public or private [5]. In the year 2023 alone, a total of 1,868 Silver Code procedures were documented, primarily in provinces situated along the coastal region such as Guayas, Manabí, Esmeraldas, and Los Ríos [6]. These challenges have presented a rigorous examination of the healthcare system’s efficacy, necessitating endeavors to enhance both quality and accessibility, alongside addressing issues of corruption and instability within medical and administrative realms.

Such challenges have resulted in disruptions to healthcare services, impacting the accessibility and continuity of care, particularly for vulnerable demographics including the elderly, pregnant women, individuals with chronic ailments, and those with disabilities. To alleviate these difficulties, a range of strategies have been implemented, notably the heightened utilization of telehealth approaches. Public institutions like the Hospital for Comprehensive Care of the Elderly in Quito have introduced teleconsulting services, drawing upon the lessons learned during the COVID-19 pandemic. This approach has proven effective in diminishing absenteeism among elderly patients, thereby ensuring uninterrupted care during periods of violence [7].

The imperative for transitioning towards a resilient healthcare system is paramount in effectively addressing public health challenges and sustaining essential healthcare services in settings marked by conflict and violence which is relatively new in the Latin America region [8]. These strategies ought to be embraced and scrutinized within analogous contexts to uphold universal health coverage and amplify the efficiency of healthcare provisions.
